# Evaluation of Surgical Outcomes of Abdominal Radical Hysterectomy and Total Laparoscopic Radical Hysterectomy for Cervical Cancer: A Retrospective Analysis of Data Collected before the LACC Trial

**DOI:** 10.3390/ijerph192013176

**Published:** 2022-10-13

**Authors:** Basilio Pecorino, Maria Gabriella D’Agate, Giuseppe Scibilia, Paolo Scollo, Andrea Giannini, Mariano Catello Di Donna, Vito Chiantera, Antonio Simone Laganà

**Affiliations:** 1 Maternal and Child Department, Obstetrics and Gynecology, Cannizzaro Hospital, University of Enna “Kore”, 95126 Catania, Italy; 2 Obstetrics and Gynecology, “Giovanni Paolo II” Hospital, 97100 Ragusa, Italy; 3 Department of Medical and Surgical Sciences and Translational Medicine, PhD Course in “Translational Medicine and Oncology”, Sapienza University, 00185 Rome, Italy; 4 Unit of Gynecologic Oncology, ARNAS “Civico-Di Cristina-Benfratelli”, 90127 Palermo, Italy; 5 Department of Surgical, Oncological and Oral Sciences (Di.Chir.On.S.), University of Palermo, 90133 Palermo, Italy; 6 Department of Health Promotion, Mother and Child Care, Internal Medicine and Medical Specialties (PROMISE), University of Palermo, 90133 Palermo, Italy

**Keywords:** cervical cancer, abdominal radical hysterectomy, total laparoscopic radical hysterectomy, minimally invasive surgery, surgical outcomes

## Abstract

Although a surgical approach is one of the key treatments for stages IA1-IIA2, results of the Laparoscopic Approach to Cervical Cancer (LACC) published in 2018 radically changed the field, since minimally invasive surgery was associated with a four-fold higher rate of recurrence and a six-fold higher rate of all-cause death compared to an open approach. We aimed to evaluate surgical outcomes of abdominal radical hysterectomy (ARH) and total laparoscopic radical hysterectomy (TLRH) for cervical cancer, including data collected before the LACC trial. In our retrospective analysis, operative time was significantly longer in TLRH compared to ARH (*p* < 0.0001), although this disadvantage could be considered balanced by lower intra-operative estimated blood loss in TLRH compared with ARH (*p* < 0.0001). In addition, we did not find significant differences for intra-operative (*p* = 0.0874) and post-operative complication rates (*p* = 0.0727) between ARH and TLRH. This was not likely to be influenced by age and Body Mass Index, since they were comparable in the two groups (*p* = 0.0798 and *p* = 0.4825, respectively). Finally, mean number of pelvic lymph nodes retrieved (*p* = 0.153) and nodal metastases (*p* = 0.774), as well as death rate (*p* = 0.5514) and recurrence rate (*p* = 0.1582) were comparable between the two groups. Future studies should be aimed at assessing whether different histology/grades of cervical cancer, as well as particular subpopulations, may have significantly different outcomes using minimally invasive surgery or laparotomy, with or without neoadjuvant chemotherapy.

## 1. Introduction

Cervical cancer is the fourth most common cancer, and one of the leading causes of death in women worldwide [1,2]. In addition, cervical cancer is a major cause of cancer deaths among women of low and middle income countries [3,4].

Surgery is one the key therapies applied during the early International Federation of Gynecology and Obstetrics (FIGO) stage, including stage IA1 with lymphovascular space invasion (LVSI), IA2, IB1, IB2, IB3, IIA1, and IIA2 cervical cancer [5]. Abdominal radical hysterectomy (ARH) has been a classic landmark surgical procedure of gynecologic oncologist for cervical cancer [6], although early-stage cervical cancer surgery can be managed by minimally invasive surgery, which is associated with lower intra-operative morbidity and faster recovery compared with laparotomy and allows an accurate nerve-sparing approach [7].

The Laparoscopic Approach to Cervical Cancer Trial (LACC trial) radically changed the surgical treatment of early-stage cervical cancer [8]. This trial included patients with stage IA1 LVSI, IA2, or IB1 cervical cancer and a histologic subtype of squamous-cell carcinoma, adenocarcinoma, or adenosquamous carcinoma, and randomly assigned patients to undergo minimally invasive surgery or open surgery. As is widely known, this trial was stopped early by the data and safety monitoring committee after enrolling 631 of a planned 740 patients. The disease free survival rate at 4.5 years was 86% among women assigned to minimally invasive surgery and 96.5% in those who underwent open surgery. Minimally invasive surgery was also associated with inferior overall survival [9]. Retrospective analysis using National Cancer Institute (NCI)’s Surveillance, Epidemiology, and End Results (SEER) data in the US confirmed the results of the LACC trial, demonstrating that minimally invasive surgery was associated with an increase in mortality rate in patients affected by cervical cancer [10].

We aimed to evaluate both surgical outcomes of ARH and total laparoscopic radical hysterectomy (TLRH) for cervical cancer with a retrospective analysis of data collected before the LACC trial.

## 2. Materials and Methods

This is a retrospective analysis of surgical cases performed before the LACC trial, collected between January 2013 and May 2018, after approval by the independent Institutional Review Board of the Cannizzaro Hospital (ID: 34/2021). The design, analysis, interpretation of data, drafting, and revisions conform to the Helsinki Declaration, the Committee on Publication Ethics guidelines (http://publicationethics.org/), and the Strengthening the Reporting of Observational Studies in Epidemiology Statement [11], available through the Enhancing the Quality and Transparency of Health Research Network (www.equator-network.org). The data collected were anonymized, taking into account the observational nature of the study, without personal data that could lead to formal identification of the patient. Each patient enrolled in this study was informed that the procedure could be converted to laparotomy, if necessary, and signed a consent to allow data collection and analysis for research purposes. The study was not advertised. No remuneration was offered to the patients to give consent to be enrolled in this study.

The inclusion criteria were cervical cancer defined as FIGO stage IA1-IVB. Cervical cancer patients were treated depending on stage of disease according to the 2013 Italian Association of Medical Oncology (AIOM) guidelines, the 2017 National Comprehensive Cancer Network (NCCN) guidelines [12], and the American Society of Clinical Oncology Resource-Stratified Clinical Practice Guidelines [13]. All patients who underwent radical hysterectomy were considered eligible, irrespective of demographic characteristics, histological features, the surgical access route, and if the patient underwent surgery as the primary treatment (upfront) or after neoadjuvant chemotherapy (NACT).

Patients with FIGO stage ≥ IB2-IIB FIGO, defined as locally advanced cervical cancer, underwent NACT after the evaluation of Performance Status Scores, as based on the Eastern Cooperative Oncology Group (ECOG) scale which ranges from 0 to 4, with higher values indicating greater disability [14]. NACT was administered as TIP (intravenous ifosfamide 5000 mg/m^2^ and mesna intravenous 5000 mg/m^2^ on day 1, intravenous paclitaxel 175 mg/m^2^ and intravenous cisplatin 75 mg/m^2^ on day 2, every three weeks for three cycles) or CBDCA + PTX protocol (3 cycles of paclitaxel 175 mg/m^2^ and carboplatin AUC 5–6 every 3 weeks), as previously described [15].

An accurate clinical evaluation after NACT was mandatory. We measured the response to NACT performing a pelvic examination plus pelvic magnetic resonance imaging (MRI) before and after NACT, in accordance with the Response Evaluation Criteria in Solid Tumors (RECIST) guideline [16]. Patients with optimal response (OPR), defined as a complete pathological response (CPR) with tumor disappearance in the cervix and negative nodes or a residual disease with <3 mm stromal invasion, as well as suboptimal response (SOR) patients, defined as persistent residual disease with >3 mm stromal invasion on surgical specimen (pPR2), underwent radical hysterectomy and pelvic lymphadenectomy within 4 weeks of the last chemotherapy cycle, while patients without a clinical response, defined as <50% decrease or <25% increase in the two largest perpendicular diameters of the measurable lesion, were managed case by case after a multidisciplinary meeting discussion.

In all patients, surgery was preceded by the same preoperative work for staging purposes, including routine blood tests, oncological markers dosage, chest X-ray, electrocardiogram, pelvic and lower abdomen MRI, positron-emission tomography (PET) and pelvic ultrasound scan. A computerized axial tomography (CT) was also performed, when indicated. An anesthesiologist evaluated all women scheduled for surgery, to define the risk class and assign the American Society of Anesthesiologists (ASA) physical status score. Radical hysterectomies were performed by expert onco-gynecologists, as classified by Querleu and Morrow [17]. During TLRH, a Rumi II (Cooper Surgical, Trumbull, USA) uterus manipulator was routinely positioned. Colpotomy was performed intracorporeally with the help of a colpo-pneumo occluder, and colporraphy was performed laparoscopically with single stitches. Pelvic lymphadenectomies were carried out as type II dissections according to the Cibula and Abu-Rustum’s classification [18], i.e. removal of nodes anterior and medial to the external iliac vessels, including those caudal in the “Leveuf and Godard area” (interiliac), nodes between external iliac vessels and psoas muscle after skeletonization of vessels, ventrally, distal nodes caudal to the deep circumflex iliac vein, internal iliac nodes, anterior nodes up to the mid common iliac vessels, nodes below the obturator nerve, and presacral nodes.

An ASA Score ≥ IV was considered a contraindication to surgery. Perioperative antibiotics were routinely administered to prevent surgical site infections. Low molecular weight heparins (LMWH) were administered depending on women’s weight and the patient-specific score calculated on the hospital protocol chart. Ward gynecologists and nurses were committed for postoperative patient management, except in those intervals during which women were sent to the intensive care unit (ICU) for the first few days after surgery, when needed. A bladder catheter removal was planned for the third post-operative day, with subsequent postminctional urinary volume evaluation, and intermittent catheterization or self-catheterization training in cases when post-voiding residue was greater than 100 mL.

Adjuvant pelvic radiotherapy, or chemotherapy and radiotherapy, was administered at the discretion of a multidisciplinary team consisting of onco-gynecologists, oncologist, pathologists and radiotherapist, generally prescribed after stratification for the following risk factors: positive pelvic lymph nodes, parametrial invasion, positive parametrial and/or vaginal margins (high risk factors) and according to the Sedlis criteria (intermediate risk factors) based on cervical stromal invasion, LVSI and tumor size [19]. Adjuvant radiotherapy was offered to patients with two or more intermediate risk factors, whereas platinum-based adjuvant concurrent chemo-radiotherapy was offered to patients with one or more high risk factors [20]. Radiotherapy consisted of pelvic external beam radiation (EBRT) with a dose of 50.4 Gy over 5–6 weeks using daily fractions of 1.7–1.8 Gy for 5 days/week setting the upper border of the radiation field at the L4/L5 interspace. Patients with positive surgical margins or parametrial involvement received a further boost dose (10–14 Gy) on the lower pelvis.

Patients underwent follow-up every 3 months for the first two years, every 6 months during the next three years and yearly thereafter. The treatment of recurrence depended on site, the patient’s performance status, and previous cervical cancer treatment.

Statistical analysis was performed with InStat 3.10, GraphPad software, San Diego, CA, USA. Continuous variables were expressed as mean and standard deviation (SD). Categorical variables were expressed as frequency and percentage. The independent t test and Wilcoxon rank-sum test were used to compare continuous variables as appropriate. The χ2 test and Fisher’s exact test were used to compare categorical data. A *p* value < 0.05 was considered statistically significant.

## 3. Results

We screened 196 patients with cervical cancer. Among this population, 151 women underwent surgical treatment, 72 were treated with ARH and pelvic lymphadenectomy, and 40 with TLRH and pelvic lymphadenectomy (radicality is reported in Table 1).

Main demographic characteristics are reported in Table 2. The two groups did not show significant differences in age (*p* = 0.0798), Body Mass Index (*p* = 0.4825) or parity (*p* = 0.5189).

Patients’ distribution for FIGO stages is shown in Table 3. Most women had a stage IIB disease in both cohorts. The squamous histotype was the most frequent, being diagnosed in 79.2% of ARH cases and in 57.5% of TLRH cases.

We did not find significant differences in the mean number of pelvic lymph nodes retrieved (21.7 ± 11.9 in ARH and 22.3 ± 8.7 in TLRH; *p* = 0.153) and nodal metastases (35 in ARH and 16 in TLRH; *p* = 0.774).

Among the total population of women who underwent surgery, 92 (60.9%) were under 43 years old. In 26 women (28.3%) bilateral ovarian preservation was feasible. In nine of them (9.8%), at least a single ovary was spared.

Surgical outcomes as well as intra- and post-operative complications are reported in Table 4.

Operative time was significantly shorter (*p* < 0.0001) in ARH (129.6 ± 25 min) compared with TLRH (242.7 ± 72.9 min). Conversely, estimated blood loos was significantly higher (*p* < 0.0001) in ARH (246.8 ± 113.6 cc) compared with TLRH (152 ± 72.9 cc). We did not find significant differences for the number of transfusions (*p* = 0.4863), hospital stay (*p* = 0.1529), intra-operative (*p* = 0.0874) and post-operative complication rate (*p* = 0.0727). Of note, we included and reported only complications occurring within 60 days following the surgery, excluding long-term complications associated with adjuvant radiotherapy.

Among 196 cervical cancer patients, NACT was administered in 79 women (40.3%), 16 (40%) of whom underwent TLRH, while 24 (60%) underwent ARH.

At a median follow-up of 33 months (Table 5), 18 (25%) of ARH patients and 38 (95%) of TLRH patients had been followed-up. Three deaths (one of which happened as an early post-operative complication) and five recurrences, occurred only in the ARH group, while no recurrences were found in the TLRH group. We did not find significant differences for death rate (*p* = 0.5514) or recurrence rate (*p* = 0.1582) among the patients who were followed-up.

## 4. Discussion

Surgical management of cervical cancer underwent a significant paradigm shift after the results of the LACC trial became available [21]. Despite the known advantages of minimally invasive surgery, worse oncological outcomes using this approach made it improper for the management of cervical cancer. Another controversial point was the complication rate using open surgery or minimally invasive surgery for this purpose. On the one hand, some authors hypothesized that minimally invasive surgery could best fit some particular population such as non-obese women, whereas on the other hand, age was considered a risk factor by some authors [22,23], and other suggested that age was not a barrier to radical surgery in patients with early-stage cervical cancer [24]. In our series, we did not find significant differences for intra-operative (*p* = 0.0874) and post-operative complication rate (*p* = 0.0727) between ARH and TLRH. This is not likely to be influenced by age and Body Mass Index, since they were comparable in the two groups (*p* = 0.0798 and *p* = 0.4825, respectively). From this point of view, laparoscopy allows reduced peritoneal damage, thus resulting in less postoperative adhesion formation between the anterior abdominal wall and intraperitoneal structures, which is of important benefit if adjuvant radiotherapy is indicated, with a subsequent decrease of toxicity [25]. This may be of paramount importance, considering that an open approach was found to be the main morbidity predictor for patients undergoing radical hysterectomy plus adjuvant radiotherapy [26].

In our retrospective analysis, operative time was significantly longer in TLRH compared with ARH (*p* < 0.0001), although this disadvantage could be considered balanced by the lower intra-operative estimated blood loss in TLRH compared with ARH (*p* < 0.0001), confirming previous findings [6,25]. In addition, performances during laparoscopic or laparotomic lymphadenectomies were comparable. Indeed, we did not find significant differences for the mean number of pelvic lymph nodes retrieved (*p* = 0.153) and nodal metastases (*p* = 0.774) between the two groups, in line with other series [27].

Several limitations of our study should be considered for proper data interpretation. First, the retrospective nature of the study can be considered an intrinsic bias. Second, we did not perform a sub-analysis for different histology and grading of cervical cancer. Third, the follow-up was relatively short, and a quite high number of women were lost-to-follow-up in the ARH group. Fourth, our setting was biased by the propensity to manage reversible complications that sometimes occurred after radical interventions (such as temporary urinary retention) in the hospital, rather than on an outpatient setting after hospital discharge. Fifth, the group analyzed is heterogeneous, including a high number of patients with large tumors who underwent prior chemotherapy for subsequent evaluation of surgical treatment: in particular, this type of patient could not be considered an adequate population to assess *per se* the advantages or disadvantages of a surgical approach, as it is a population with an uncertain prognosis and factors with a worse prognosis. Finally, the relatively low number of enrolled women further reduces the power of the conclusions of this study. Nevertheless, this can be considered one series among several others published, but this can contribute to current knowledge about the management of the disease and be added to a pooled analysis in case of a future systematic review.

Despite the short follow-up (median 33 months), we found that death rate and recurrence rate were comparable between ARH and TLRH (*p* = 0.5514 and *p* = 0.1582, respectively). In this regard, other authors found even higher disease free surgical results in the laparoscopic group and more frequent loco-regional recurrences in the laparotomic group [27]. This line of evidence may suggest that the use of a manipulator (and consequent risk of uterine perforation), Trendelenburg position and intracorporeal colpotomy under CO_2_ pneumoperitoneum, which are believed to increase the risk of recurrence and play a detrimental role on disease free survival and overall survival in women undergoing minimally invasive surgery, could be further re-evaluated in future analysis for a proper risk assessment.

Accumulating evidence suggests adequate outcomes of laparoscopic surgery in women affected by locally advanced cervical cancer (stage IB2-IIB) undergoing NACT followed by surgery [28]. However, at the same time, radical hysterectomy after NACT can be technically challenging due to the desmoplastic reaction, and loss of normal dissection planes, as a result of the cytotoxic treatment [29].

Finally, future research should further investigate the role of robotic surgery, which was associated with oncological outcomes similar to open surgery in the recently published MEMORY Study [30], and to perform sub-analyses of surgical/oncological outcomes according to different histologies of cervical cancer [31] managed using ARH or TLRH.

## 5. Conclusions

Results of the LACC trial subverted current knowledge, forcing any oncology center to an internal audit and a reflection about the appropriateness of care offered. The conflicting data should be discussed with patients, underlying that future evidence may change current clinical practice, but at the same time the patients’ chance of survival must not be affected by inadequate surgery, even if technologically more attractive.

Since cervical cancer is not a single disease, future studies should assess whether different histology/grade of cervical cancer, as well as particular subpopulations, may have significantly different oncological outcomes using minimally invasive surgery or laparotomy, with or without previous NACT.

## Figures and Tables

**Table 1:** Radicality of hysterectomy in women who underwent abdominal radical hysterectomy (ARH) and total laparoscopic radical hysterectomy (TLRH) for cervical cancer.

Radicality ^1^	ARH, *n* (%)	TLRH, *n* (%)
A	1 (1.4)	0 (0)
B1	1 (1.4)	4 (10)
B2	0 (0)	4 (10)
C1	62 (86.1)	26 (65)
C2	6 (8.3)	0 (0)
Mixed	2 (2.8)	6 (15)
Total	72 (100)	40 (100)

^1^ Radical hysterectomies, classified according to Querleu and Morrow [17].

**Table 2:** Demographic characteristics of women who underwent abdominal radical hysterectomy (ARH) and total laparoscopic radical hysterectomy (TLRH) for cervical cancer.

Parameter	ARH (*n* = 72)	TLRH (*n* = 40)	*p*
Age (mean ± SD)	53.1 ± 12.2	48.9 ± 11.9	0.0798
Body Mass Index (Kg/m^2^)	26.2 ± 4.9	25.5 ± 5.1	0.4825
Parity	2.1 ± 1.5	2.3 ± 1.6	0.5189

**Table 3:** Stage, histology and grade of the cervical cancer in women who underwent abdominal radical hysterectomy (ARH) and total laparoscopic radical hysterectomy (TLRH).

Stage	ARH, *n* (%)	TLRH, *n* (%)
IA1	1 (1.4)	/
IA2	1 (1.4)	5 (12.5)
IB1	16 (22.2)	15 (37.5)
IB2	5 (6.9)	2 (5)
IIA	4 (5.6)	/
IIB	45 (62.5)	18 (45)
**Histology**		
Squamous cell carcinoma	57 (79.2)	23 (57.5)
Adenocarcinoma	12 (16.7)	11 (27.5)
Adenosquamous	1 (1.4)	1 (2.5)
Not specified	1 (1.4)	5 (12.5)
**Grade**		
G1	0 (0)	1 (2.5)
G2	19 (26.4)	11 (27.5)
G3	13 (18.1)	18 (20)
Not specified	40 (55.6)	19 (47.5)

**Table 4:** Surgical, intra-operative and post-operative complications in women who underwent abdominal radical hysterectomy (ARH) and total laparoscopic radical hysterectomy (TLRH) for cervical cancer.

Surgical Variables	ARH (*n* = 72)	TLRH (*n* = 40)	*p*
Operative time (min)	129.6 ± 25	242.7 ± 72.9	<0.0001
Estimated blood loss (cc)	246.8 ± 113.6	152 ± 72.9	<0.0001
Transfusions, *n* (%)	7 (9.7)	2 (5)	0.4863
Length of hospital stay (days)	6 ± 2.7	5.25 ± 2.6	0.1529
**Intra-operative complications, *n* (%)**			
Vascular injuries	8 (11.1)	7 (17.5)	
Detrusor fibers disjunction	2 (2.8)	2 (5)	
Uterus perforation by manipulator	/	1 (2.5)	
Bowel injury	1 (1.4)	0 (0)	
Conversion to laparotomy	/	2 (5)	
Total	11 (15.2)	12 (30)	0.0874
**Post-operative complications, *n* (%)**			
Re-laparotomy	2 (2.8)	/	
Cardiac arrest	1 (1.4)	0 (0)	
Death	1 (1.4)	0 (0)	
Fever	4 (5.6)	2 (5)	
Anemia	2 (4.2)	1 (2.5)	
Intraoperative transfusions	2 (2.8)	1 (2.5)	
Postoperative ileus	2 (2.8)	1 (2.5)	
Subcutaneous emphysema	/	1 (2.5)	
Laparotomy wound dehiscence	1 (1.4)	/	
Vaginal cuff dehiscence	0 (0)	2 (5)	
Vaginal leakage	0 (0)	1 (2.5)	
Lymphocele/lymphocyst	4 (5.6)	3 (7.5)	
Vesico-vaginal fistula	1 (1.4)	0 (0)	
Ureter-vaginal fistula	1 (1.4)	2 (5)	
Entero-vaginal fistula	1 (1.4)	2 (5)	
Entero-cutaneous fistula	1 (1.4)	0 (0)	
Thigh paresthesia	0 (0)	1 (2.5)	
Voiding sequelae from denervation	3 (4.2)	5 (12.5)	
Total	26 (36.1)	22 (55%)	0.0727

**Table 5:** Follow-up in women who underwent abdominal radical hysterectomy (ARH) and total laparoscopic radical hysterectomy (TLRH) for cervical cancer.

	ARH (*n* = 72)	TLRH (*n* = 40)	*p*
Patients in follow-up (median 33 months)	18 (25)	38 (95)	
Death	3 (16.6)	0 (0)	0.5514
Recurrence of disease	5 (27.8)	0 (0)	0.1582

## Data Availability

A full dataset is available from the corresponding author on reasonable request.
